# Évaluation de la conformité de la tenue du Partogramme dans une maternité Tunisienne: audit clinique ciblé

**DOI:** 10.11604/pamj.2017.27.106.10620

**Published:** 2017-06-12

**Authors:** Latifa Merzougui, Nedia Marwen, Tarek Barhoumi, Afef Ben Ltaeifa, Hajer Hannachi, Roukaya Jaballah, Ridha Fatnassi

**Affiliations:** 1 Service d’Hygiène Hospitalière, CHU Ibn El Jazzar, Kairouan, Tunisie; 2 Service de Gynécologie Obstétrique, CHU Ibn El Jazzar, Kairouan, Tunisie

**Keywords:** Partogramme, audit clinique, évaluation des pratiques professionnelles, obstétrique, Partograph, clinical audit, evaluation of professional practices, obstetrics

## Abstract

Le partogramme est un outil incontournable dans la pratique quotidienne en salle de naissance. C'est le reflet écrit de la qualité de la prise en charge materno-foetale pendant l'accouchement et le post-partum immédiat. Le but de notre travail est d'évaluer la conformité de la tenue du partogramme au sein de notre maternité et de proposer des axes d'amélioration de la qualité de sa tenue. il s'agit d'une étude rétrospective par audit clinique, effectuée sur 400 dossiers des patientes ayant accouché dans la maternité universitaire de Kairouan du 1er Janvier au 31 décembre 2014. Le référentiel utilisé est celui de la Haute Autorité de Santé (2006) comportant 29 critères divisés en 3 domaines (PARTOten, PARTOobs, PARTOeve). le Taux de Conformité Globale (TCG) de la tenue du partogramme dans notre audit était de 55,9%. Pour le domaine I « La tenue du partogramme » (PARTOten), le TCG était de 88,9%. Pour le domaine II « la traçabilité du déroulement du travail » (PARTOobs), le TCG était de 51,4%. Pour le domaine III « la traçabilité des actes, des évènements et des traitements au cours du travail » (PARTOeve), le TCG était de 27,4%. Notre étude a permis de dégager plusieurs points à améliorer. Le but ultime de l'Audit Clinique est l'amélioration des pratiques professionnelles, de ce fait nous allons mettre en place un plan d'action (formation, sensibilisation…) qui sera suivi par une réévaluation afin de vérifier la pérennité des actions correctives.

## Abstract (en)

Partograph is an essential tool in routine delivery room practices. It is a written reflection of the quality of mother and child health management during delivery and in the immediate post-partum period. Our study aims to assess compliance rate of partograph use within our maternity ward and to propose areas for improvement of its quality. We conducted a retrospective clinical audit of 400 patients? medical records who gave birth on the labour ward at University of Kairouan from 1 January to 31 December 2014. The reference guidelines used were those from the French Health High Authority (2006) containing 29 criteria divided into 3 categories (PARTOten, PARTOobs, PARTOeve). The overall compliance rate (OCR) of partograph use in our audit was 55.9%. With regard to the first area, such as « La tenue du partogramme » (partograph use, PARTOten) the overall compliance rate was 88, 9%. With regard to the second area, such as « la traçabilité du déroulement du travail » (track labor progression, PARTOobs) the overall compliance rate was 51, 4%. With regard to the third area, such as « la traçabilité des actes, des évènements et des traitements au cours du travail » (the tracing of actions, events and treatments during labor, PARTOeve) the overall compliance rate was 27.4%. Our study has helped identify several areas for improvement. The ultimate goal of this clinical audit was the improvement of professional practices. Therefore, we implemented an action plan (training, awareness raising?) followed by a review aimed to verify the continuity of the corrective actions.

## Introduction

Selon l'Organisation Mondiale de la santé (OMS) : « le partogramme est l'enregistrement graphique des progrès du travail et des principales données sur l'état de la mère et du foetus » [1]. Dans le cadre de son programme de santé maternelle et maternité sans risque et dans le but d'uniformiser les différents graphiques et afin d'en généraliser rapidement leur adoption, l'OMS a établi en 1988 un modèle de partogramme inspiré de nombreux travaux [2, 3]. C'est un outil clé pour le suivi de l'évolution du travail et d'accouchement. Il permet la synthèse des éléments de la surveillance materno-foetale et de détecter précocement les anomalies du travail, facilite la prise de décision et la communication entre les professionnels [4].Par ailleurs il s'agit d'un document médico-légal, d'un support pour l'enseignement, pour la recherche clinique et l'évaluation des pratiques professionnelles. Par conséquent, la qualité de sa tenue est primordiale. Nos objectifs étaient d'évaluer la conformité de la tenue du partogramme au sein de la maternité du CHU Ibn El Jazzar de Kairouan et de proposer des axes d'amélioration de la qualité de sa tenue.

## Méthodes

**Figure 1:**
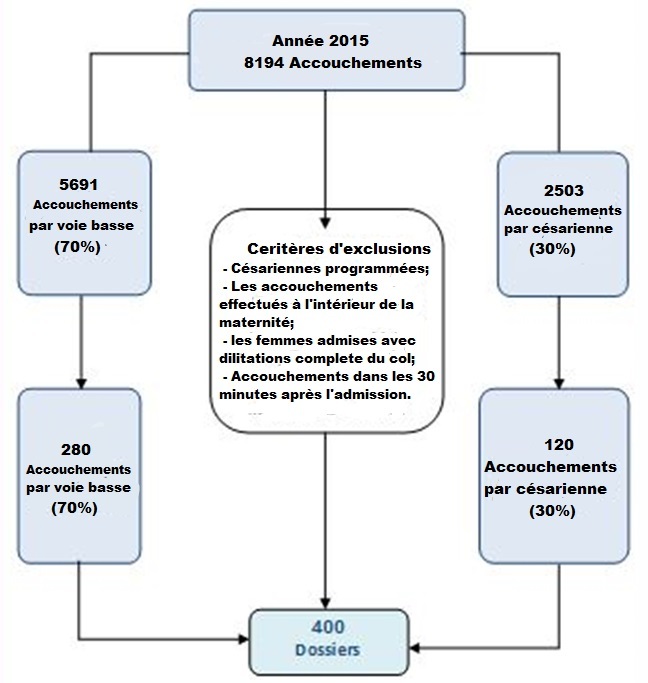
Échantillonnage

Il s'agit d'une étude descriptive rétrospective par Audit Clinique suivant la méthodologie de la HAS [5], sur un échantillon de dossiers médicaux des patientes ayant accouché dans la maternité du CHU Kairouan-Tunisie durant l'année 2014. Pour choisir notre échantillon d'étude On a stratifié notre population sur le mode d´accouchements; Ces dossiers ont été tirés au sort par un sondage aléatoire simple avec un pas de sondage 1/20. Ils ont été exclus de ce travail les césariennes programmées ou effectuées avant le début du travail, ainsi que les accouchements effectués à l'extérieur de la maternité, les femmes admises avec dilatation complète du col utérin et les femmes ayant accouché dans les 30 minutes après l'admission (Figure 1). Le référentiel utilisé est celui de la HAS en 2006 [6] comportant 29 critères divisés en 3 domaines : la tenue du partogramme : PARTOten (6 critères) ; la traçabilité du déroulement du travail : PARTOobs (12 critères) ; la traçabilité des actes, des évènements et des traitements au cours du travail : PARTOeve (11 critères). L'analyse des résultats est basée sur l'obtention du taux de conformité. Ce taux de conformité est représenté par le pourcentage de “oui” et un critère sera considéré comme conforme si son taux de conformité est supérieur à 75%.

## Résultats

**Figure 2:**
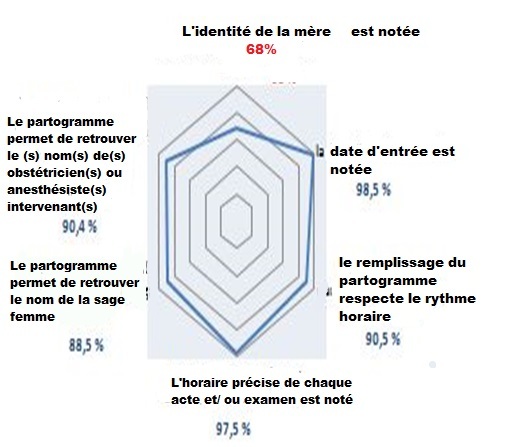
Taux de conformité par critère du domaine I (PARTOten) « la présentation du partogramme », Maternité kairouan 2014

**Figure 3:**
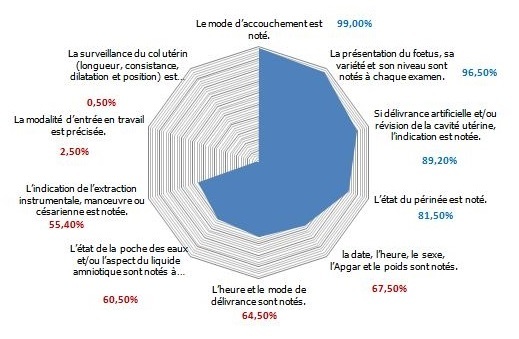
Taux de conformité par critère du domaine II ( PARTOobs) « la traçabilité du déroulement du travail »,Maternité kairouan 2014

**Figure 4:**
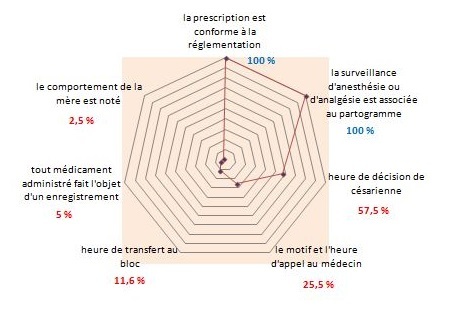
Taux de conformité par critère du domaine III (PARTOeve) « la traçabilité des actes, des événements et des traitements au cours du travail», Maternité kairouan 2014

Le total des accouchées en 2014 était de 8194 dont 2503 accouchements par césarienne soit (30%) et 5691 accouchements par voie basse soit (70%). Notre échantillon était composé de 400 dossiers médicaux: 280 accouchements par voie basse (70%) et 120 accouchements par césarienne (30%). Le taux de conformité global de la présentation du partogramme (PARTOten) était de 88,9%. Seul le critère concernant l'identité civile de la mère était non conforme (68%) (Figure 2). Le taux de conformité global du domaine II (PARTOobs) était de 51,4%. Huit critères parmi 12 étaient non conformes. En effet le taux de conformité était trop faible pour la modalité d'entrée en travail (2,5%), la surveillance du col utérin (0,5%) voir nuls pour les critères 4 « RCF est commenté à chaque examen » et 6 « le début des efforts expulsifs est noté ». Par contre, la surveillance foetale (sa présentation, sa variété et son niveau), le mode d'accouchement, l'indication de la délivrance artificielle ou de la révision utérine et l'état du périnée étaient très bien appliqués, ils ont eu des taux de conformité supérieur à 81% (Figure 3). Le taux de conformité global du domaine III (PARTOeve) était de 27,4%. La majorité des critères n'étaient pas conformes avec des taux de conformité ne dépassant les 57,5%. Ainsi plusieurs critères ont des taux de conformité nuls tel que « les paramètres cliniques sont notés à l'entrée », « l'intensité de la douleur est évaluée », « l'heure du sondage urinaire » et « la surveillance post natale ». Par contre les deux critères: « la prescription médicamenteuse est conforme à la réglementation » et « la surveillance d'anesthésie ou d'analgésie est associé au partogramme » avaient une conformité de 100% (Figure 4). Le Taux de Conformité Global de la tenue du partogramme dans notre Audit était de 55,9%. Seul le domaine I était conforme (TCG 88,9%) alors que le domaine II et III avaient un TCG respectivement de 51,4% et 27,4%.

## Discussion

L'évaluation des Pratiques Professionnelles s'inscrit dans une dynamique globale d'amélioration de la qualité et de la sécurité des soins. Elle se définit par la HAS comme étant une démarche organisée d'amélioration des pratiques, consistant à comparer régulièrement les pratiques effectuées et les résultats obtenus, avec les recommandations professionnelles. Elle vise à promouvoir la qualité, la sécurité, l'efficacité et l'efficience des soins [1]. Dans notre travail, nous avons adopté la méthode par comparaison à un référentiel (l'Audit Clinique). Il s'agit d'une méthode de diagnostic orientée vers l'action. Sa principale caractéristique est de mesurer, à l'aide de critères déterminés, les écarts entre la pratique observée et la pratique attendue (exprimée dans les recommandations professionnelles) avec l'objectif de les améliorer [5]. Le domaine I « Tenue du partogramme (PARTOten)» regroupe six critères ciblés principalement sur l'identité de la patiente et sur l'équipe l'ayant pris en charge ainsi que sur l'importance des dates et des horaires. Tous les critères de ce domaine étaient conformes sauf le critère concernant l'identité de la mère avec un taux de conformité de 68%. Ce même taux de conformité était retrouvé par l'étude de la HAS 2006 lors de la première évaluation. Alors que ce taux était trop élevé dans plusieurs autres études notamment l'étude de Manel Limem et aL [7]. en 2014, menée dans la maternité du CHU Farhat Hached de Sousse (99,3%), et dans l'étude de Anis Sghaier et al [8]. en 2011 (90%). Il était de 100% dans l'étude de Friha Soraya en 2013 réalisée dans le CHU d'Argenteuil [9] et l'étude de Provost Anne-Gaëlle en 2014 dans le CHU d'Ayres de Menezes [10] (Tableau 1).

**Tableau 1:** Conformité de la tenue du partogramme : PARTOten (revue de la littérature)

	Etudes Internationales								Etudes Nationales		
Auteur	HAS 1ère évaluation	Malibeau Claire	Catherine Boivent et *al.*	Fabien Doreus et *al.*		Marion Mottier et *al.*	Soraya Friha et *al.*	Anne.G Provost et *al.*	Anis Sghaier et *al.*	Manel Limem et *al.*	**Notre étude**
Pays	France	France Nantes	France Nantes	France		France Angers	France Argenteuil	Portugal Ayres de Menezes	Tunisie Beni Khedache	Tunisie Sousse	**Tunisie Kairouan**
				Maternité *	Maternité **						
Référence	6	13	12	11		14	9	10	8	7	-
Année	2006	2007	2009	2011		2012	2013	2014	2011	2010	2014
Taille de l'échantillon	81	122	97	90		272	109	40	120	400	400
**1.** L’identité civile de la mère est notée	68,00	99,00	99,00	97,00	74,00	94,00	100	100	90	99,30	68,00
**2.** La date est notée	88,50	88,00	100	87,00	67,00	98,00	93,00	100	93,30	99,80	**98,50**
**3.** Le remplissage du partogramme respecte le rythme horaire	89,20	74,00	64,00	16,00	13,00	88,00	99,00	17,50	68,30	84,30	**90,50**
**4.** L’horaire précis de chaque acte et/ou examen est noté	83,90	99,00	-	84,00	82,00	87,00	03,00	85,00	-	99,30	**97,50**
**5.** Le partogramme permet de retrouver le nom de(s) la sage(s) femme(s)	78,50	98,00	100	97,00	17,00	58,00	94,00	97,50	98,30	100	**88,50**
**6.** Le partogramme permet de retrouver le(s) nom(s) de(s) obstétricien(s) et/ou anesthésiste(s) intervenant(s)	76,70	-	-	62,00	27,00	-	97,00	15,00	-	82,04	**90,40**

* Maternité de Saint-Vincent-de –Paul au sein du groupe hospitalier Cochin-Saint-Vincent-de-Paul

** Maternité de Port- Royal au sein du groupe hospitalier Cochin-Saint-Vincent-de-Paul

Le domaine II associe douze critères ayant pour but d'améliorer la traçabilité et la continuité des soins. Nous avons constaté que huit critères (C1, C3, C4, C5, C6, C8, C9, C12) sur douze n'étaient pas conformes. En effet le taux de conformité de la modalité d'entrée en travail est trop bas (2,5%). En fait, dans le dossier obstétrical, on demande de préciser « l'heure du début du travail » sans préciser ni la modalité ni l'indication en cas de déclenchement artificiel. En se comparant à la littérature, ce taux variait entre 17% et 100%. Il était de 55,3% dans l'étude menée par la HAS 2006 [6] Dans le CHU d'Argenteuil, le taux de conformité de ce critère était de 95% [9] et de 100% dans les maternités du CHU de Sousse [10] et de Saint-Vincent-de-Paul [11]. Il était de 74% dans le CHU de Nantes sur des partogrammes manuscrits, et de 17% sur des partogrammes informatisés [12, 13]. Le taux de conformité de l'examen du col utérin était aussi très effondré (0,5%) car la consistance et la position étaient rarement mentionnées. La conformité de ce critère était aussi faible dans la plupart des études : elle était de 58,8% dans l'étude de la HAS 2006 [5], de 69,8% dans l'étude de Sousse [7], de 57% dans le CHU d'Argenteuil [9]. Quant à l'interprétation du RCF, la conformité était nulle car aucun tracé n'était commenté dans notre échantillon. Par contre, les tracés pratiqués au cours du travail étaient agrafés au dossier obstétrical. Cette conformité était aussi très faible dans le travail de la maternité du CHU Farhat Hached avec un taux de 9,5% [7]. Il était de 68% dans l'étude de la HAS [6]. Cependant les études d'Anis Sghaier et al. [8]; de Soraya F et al. [9];de Boivent C et al. [12]; de Malibeau C et al. [13] et de Marion Mottier et al [14] ont retrouvés des taux de conformité très satisfaisant > à 90 % (Tableau 2). Le domine III comporte onze critères visant au progrès de la traçabilité des actes, évènements marquants et traitements administrés au cours du travail et du post-partum immédiat concernant la mère et l'enfant. Seulement deux critères étaient conformes avec un taux de conformité de 100% : «la prescription médicamenteuse est conforme à la réglementation» et «la fiche d'anesthésie est associée au partogramme». Alors que les critères qui portent sur « la surveillance maternelle : les paramètres cliniques à l'entrée », « le comportement de la mère », « l'intensité de la douleur et la surveillance post natale », n'étaient pas conformes. En effet le taux de conformité du critère 1 « les paramètres cliniques à l'entrée » était nul à cause de l'absence de la pulsation. Dans l'étude de Limam et al [7] , la pulsation n'était notée que dans 15,3% des dossiers, par contre la pression artérielle et la température étaient précisées dans la plupart des dossiers. Ce critère était également non conforme (1% à 2%) dans plusieurs autres études [11]. La pulsation était aussi négligée dans la surveillance post natale ce qui donne encore une conformité nulle.

**Tableau 2:** Conformité de la traçabilité des actes, des évènements et des traitements au cours du travail : PARTOeve (revue de la littérature)

Auteur	HAS 1^ère^évaluation	Malibeau Claire.	Catherine Boivent et *al.*	Fabien Doreus et al	Marion Mottier et *al.*	Soraya Friha et *al.*	Anne.G Provost et *al.*
Référence	6	13	12	11	14	9	10
**1.** les paramètres cliniques sont notés à l’entrée (PA, pools, température)	37,40	-	25,00	1,00	-	55,00	95,00
**2.** le comportement de la mère pouvant nuire à sa sécurité est noté	41,00	5,00	58,00	47,00	94,00	46,00	7,50
**3.** La pose de la péridurale et ses réinjections sont notées	88,20	-	-	75,00	94,00	100	-
**4.** L’intensité de la douleur est évaluée	23,00	64,00	42,00	11,00	37,00	52,00	40,00
**5.** La prescription est conforme à la réglementation (nom du prescripteur, nom du médicament, posologie, voie d’administration)	42,80	-	-	21,00	-	63,00	15,00
**6.** Tout médicament administré fait l’objet d’un enregistrement	62,40	-	-	-	-	14,00	37,50
**7.** L’heure du sondage(s) urinaire(s) est notée	53,50	48,00	62,00	85,00	79,00	7,00	2,50
**8.** La surveillance d’anesthésie ou d’analgésie est associée au partogramme	60,60	-	-	-	60,00	98,00	0,00
**9.** Le motif et l’heure d’appel au(x) médecin(s) spécialiste(s) sont notés	39,20	-	52,00	33,00	-	18,00	-
**10. a.** L’heure de décision de césarienne est notée		0,00	90,00				
**10. b** l.’heure de transfert au bloc est notée	55,30	0,00	33,00	36,00		1,00	15,00
**11.** La surveillance postnatale est notée sur le partogramme	42,80	43,00	70,00	0,00	-	83,00	87,50

Nos résultats sont concordants avec celle retrouvée dans les études de la maternité de Saint-Vincent-de-Paul et de Port Royal [11]; mais inférieure aux taux retrouvés dans l'étude de la HAS[6], de Sousse[7], de Béni Khedache [8], d'Argenteuil [9] et d'Ayres de Menezes [10] dont les taux de conformité étaient respectivement de 42,8%, 85,9%, 90,8%, 83% et 87,5% (Tableau 3). Par la suite nous avons présenté nos résultats aux professionnels de la maternité de kairouan, et suite à un brainstorming, nous avons élaboré plusieurs causes des écarts pouvant expliquer la non-conformité de la tenue du partogramme tel que le manque de sensibilisation et de formation, le manque du temps dédié au remplissage lié à la surcharge du travail, le manque de cases dédiées au remplissage de plusieurs items, l'ignorance ou l'oubli de certaines recommandations professionnelles et plusieurs informations n'étaient pas notées car elles sont considérées comme évidentes. Suite à cette rencontre, nous avons opté pour les actions correctives suivantes : sensibilisation et formation des professionnels de la maternité; actualisation du partogramme pour cela nous proposons d'ajouter des items supplémentaires dans le dossier obstétrical; élaboration d'un outil de surveillance du post-partum immédiat tel qu´ une fiche de surveillance qui porte sur l'évaluation du volume des pertes sanguines, l'état du globe utérin, la fréquence cardiaque et la mesure de la tension artérielle); l'utilité de l'affichage et diffusion des recommandations des bonnes pratiques. Enfin, nous allons organiser périodiquement des visites d'évaluation formatives sur l'utilisation du partogramme et de réévaluer la qualité de sa tenue afin de s´assurer de la pérennité et de l´efficacité des mesures correctives. En effet le partogramme reste un outil toujours actuel pour évaluer la qualité des soins en obstétrique [15]. L'EPP reste, en fait, difficile à mettre en place dans les établissements de santé notamment en l'absence d'organisation et de réglementation en faveur de la mise en oeuvre des démarches qualité et sécurité des soins.

**Tableau 3:** Conformité de La traçabilité de déroulement du travail : PARTOobs (revue de la littérature)

Auteur	HAS 1^ère^évaluation	MalibeauClaire.	CatherineBoivent et *al.*	FabienDoreus et *al.*	MarionMottier et *al.*	SorayaFriha et *al.*	Anne.GProvost et *al.*
Référence	6	13	12	11	14	9	10
**1.** La modalité d’entrée en travail est précisée	55,30	74,00	17,00	100	49,00	95,00	85,00
**2.** La présentation du foetus, sa variété et son niveau sont notés à chaque examen	68,80	-	-	68,00	-	84,00	37,50
**3.** La surveillance du col utérin (longueur, consistance, dilatation) est notée à chaque examen	58,80	-	97,00	22,00	84,00	57,00	40,00
**4.** Le rythme cardiaque foetal est commenté à chaque examen	68,00	91,00	94,00	47,00	95,00	94,00	62,50
**5.** L’état et l’aspect de la PDE et/ou du liquide amniotique sont notés à chaque examen	78,40	-	98,00	37,00	-	09,00	32,50
**6.** Le début des efforts expulsifs est noté	51,70	43,00	68,00	09,00	54,00	97,00	47,50
**7.** Le mode d’accouchement est noté	96,30	-	45,00	99,00		99,00	87,50
**8.** L’indication de l’extraction instrumentale, manoeuvres ou césarienne est notée	59,70	-	-	96,00	25,00	99,00	42,50
**9.** L’heure et le mode de délivrance sont notés	47,00	30,00	72,00	23,00	78,00	92,00	82,50
**10.** Si délivrance artificielle et/ou révision de la cavité utérine, l’indication est notée	55,30	100	63,00	50,00	65,00	97,00	15,00
**11.** L’état du périnée est noté	90,10	88,00	75,00	38,00	13,00	46,00	60,00
**12.** La date, l’heure, le sexe, l’Apgar et le poids sont notés	76,70	-	-	87,00	-	98,00	72,5,

## Conclusion

L'évaluation de la tenue du partogramme constitue la première étape d'une politique d'amélioration de la qualité des soins délivrés à la mère et au foetus. Notre étude nous a permis d'évaluer la qualité de la tenue du partogramme et d'identifier les facteurs pouvant être liés à la non-conformité. Un plan d'amélioration a été mis en place. Il sera suivi par une réévaluation pour satisfaire l'obligation d'amélioration continue de la démarche qualité.

### Etat des connaissances actuelles sur le sujet

- Le partogramme reste un outil toujours actuel pour évaluer la qualité des soins en obstétrique ;
- Le taux de conformité de la tenue du partogramme est encore faible.

### Contribution de notre étude à la connaissance

- Notre étude est le premier Audit clinique qui a permis de déterminer la conformité de la tenue du partogramme dans notre Hôpital;
- Ce travail constitue une opportunité pour les professionnels pour se familiariser avec les outils de l'évaluation des pratiques professionnelles;
- A l'issu de cette étude un plan d'amélioration de la qualité de la tenue du partogramme a été mis en place.

## Conflits d’intérêts

Les auteurs ne déclarent aucun conflit d'intérêt.
